# A novel design for porphyrin based D–s–A systems as molecular rectifiers†
†Electronic supplementary information (ESI) available: Schematic representation of the electro-grafting mechanism, the electrochemical deposition of undecene, *I*–*V* characteristics of the blank Si sample and C-11 alkyl monolayers on a silicon wafer, XPS of the monolayers and the optimised geometry of the **5b** congener with reverse geometry. Tables of atom coordinates and absolute energies used for theoretical calculations. See DOI: 10.1039/c5sc03590b


**DOI:** 10.1039/c5sc03590b

**Published:** 2015-11-16

**Authors:** Kavita Garg, Chiranjib Majumder, Shiv K. Gupta, Dinesh Kumar Aswal, Sandip Kumar Nayak, Subrata Chattopadhyay

**Affiliations:** a Bio-Organic Division , Bhabha Atomic Research Centre , Mumbai , India . Email: kavitachemistry1@gmail.com ; Email: schatt@barc.gov; b Chemistry Division , Bhabha Atomic Research Centre , Mumbai , India; c Technical Physics Division , Bhabha Atomic Research Centre , Mumbai , India

## Abstract

Two Si-based hybrid self-assembled monolayers of porphyrin based on a D–s–A system were synthesized by electro-grafting. The monolayers showed a stable and reversible rectification at room temperature. The monolayer fabricated using a porphyrin with an eleven-carbon alkyl chain linker was comparatively more compact and exhibited a 10^5^ times higher rectification ratio (RR) relative to another similar system that had a six-carbon alkyl chain linker, possibly because of the compact packing.

## Abstract 2

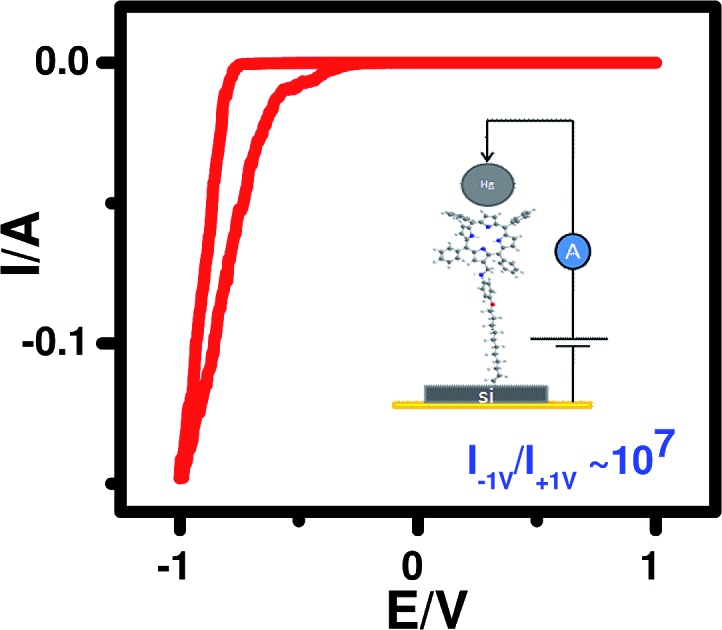
Electro-grafted porphyrin-based D–s–A monolayers on Si behave as rectifiers.

## Introduction

Miniaturization is a vital need of the electronics industry, but it is limited by changes in the bulk properties of materials as they move to nanoscale dimensions. Most of the successes in this field have focused on the electrical properties of organic molecules placed between metal electrodes.^1^–^5^ In particular, self-assembled monolayers (SAMs) of alkanes and aromatic thiols on gold substrates have been very popular for constructing metal–molecule–metal (MMM) junctions.^6^–^10^ Concurrently, efforts aimed at synthesizing metal–molecule–semiconductor (MMS) junctions by covalent linking of organic molecules to semiconductor surfaces are gaining momentum.^11^ Such assemblies present opportunities for novel molecular electronic charge transport mechanisms, and are potentially compatible with conventional metal oxide–semiconductor (MOS) technology. To this end, there is a burgeoning interest in small organic molecules capable of switching their redox status, which, in association with semi-conductors such as Si, may scale down the size of the molecular electronic devices.^12^–^14^ Here, surface potential tailoring can be achieved by chemically-grafting organic molecules onto Si to develop improved hybrid molecular devices. For example, the p–n junction threshold voltage for rectification can be adjusted by changing the electronic nature of the organic π group molecules, instead of *via* the classical doping method.^14^ Different techniques such as making Langmuir–Blodgett (LB) films^15^ or SAMs of organic molecules on solid substrates *via* MMM junctions^16^ are most commonly used for this purpose. Compared to the LB films, SAMs are easy to prepare and may be more robust as the organic molecules are sturdily anchored onto the metal substrates at fixed distances. Chemically bonded monolayers on Si surfaces can be prepared either on Si oxide (SiOx) surfaces or on oxide-free Si,^11^,^17^ the latter being preferred due to better electronic coupling of the Si molecules and the lack of charging effect. In-depth reviews with excellent analyses of the different methods of fabrication and characterization of SAM junctions on H-terminated Si surfaces are available.^18^,^19^ The protocols usually adopted for constructing densely packed Si–organic hybrids involve the deposition of functionalized alkenes/alkynes using heat,^20^ light,^21^,^22^ electrochemical techniques, radical initiators^23^ or Lewis acids,^24^ as well as alkylhalides *via* either a Grignard route or lithiation.^25^ This is followed by attachment of the electro-active organic molecules to the terminal functionality of the resultant alkane/alkene–Si hybrids by esterification or amidation. However, due to steric factors, not all of the deposited alkane/alkene moieties can be modified with organic molecules. This may produce non-uniform organic–Si hybrids. In our previous work, we found that cathodic electro-grafting of pre-synthesized alkenylated electro-active organic molecules onto a Si–H surface can conveniently provide SAM-based molecular electronics devices with the following advantages:^26^ the process is simple; it can be monitored *in situ* to ensure completion of deposition; it can exclude oxidation and/or hydrolysis at the Si surface due to the negative potential bias of the Si wafers; and it can produce materials where the Si–H surface is modified only by the chosen molecules.

Amongst the many electron-rich organic molecules, porphyrins^27^ are ideally suited for fabricating molecular devices because they: (i) can form stable π-cation radicals and exhibit two accessible cationic states in their monomeric forms;^28^–^32^ (ii) have long charge retention times, resulting in lower power consumption; (iii) are highly stable,^33^ and (iv) can form self-assembled structures.^34^ In view of these favourable attributes, porphyrins have been extensively used as π molecules for the construction of storage devices, molecular wires and memory devices. Reports on current rectification using C_60_–porphyrin combinations also exist.^35^ Molecules exhibiting rectification behavior with a high rectification ratio (RR) are very useful for making diodes. According to Aviram and Ratner, a single molecule with a donor–spacer–acceptor (D–s–A) structure should behave as a rectifying diode when placed between two electrodes, where the σ-bond bridge prevents the direct overlap of the donor (D) and acceptor (A) energy levels to allow unidirectional flow of current.^1^ Several groups have experimentally verified this model, but porphyrins have never been used for this purpose in silicon hybrid systems.^36^,^37^ In the present investigation, two such single molecules (**5a**/**5b**) were synthesized, where porphyrin and aniline moieties served as the (A) and (D) units respectively, while a –CH_2_–NH– moiety was anticipated to be a suitable spacer. These molecules were electro-grafted onto Si-surfaces using the C-6/C-11 alkenyl chain of **5a**/**5b** as the linker to construct the respective MMS heterostructures. Measurement of their *I*–*V* behavior revealed high current RRs for these assemblies. Moreover, a subtle change in the linker length significantly changed the monolayer packing on the Si-surface, resulting in a pronounced alteration in the current rectification properties.

## Results and discussion

### Synthesis of the porphyrins

**Scheme 1:**
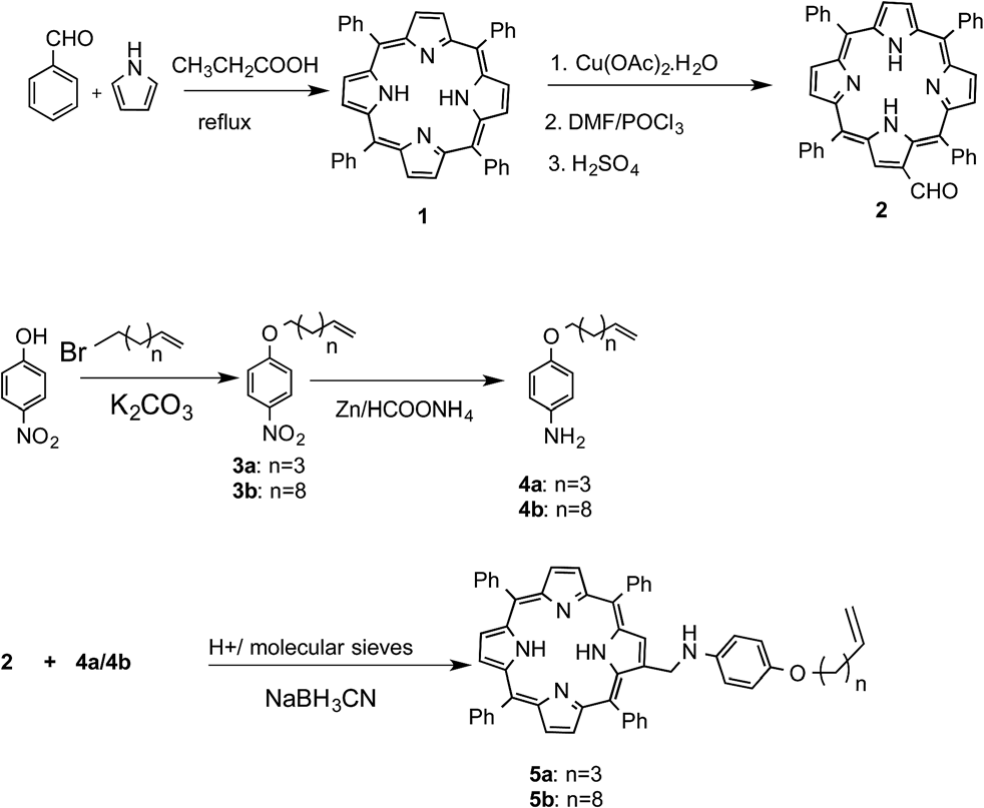
Synthesis of the D–s–A molecules **5a** and **5b**.

Porphyrin-based functional molecules are, by and large, synthesized *via* functionalization of the aryl moieties of unsymmetrical *meso*-tetraryl porphyrins. However, the synthesis of unsymmetrical porphyrins is fraught with limitations such as poor yields and tedious isolation procedures. Instead, functionalization of the pyrrole units of the porphyrins offers a better alternative to alter the porphyrin scaffold. However, this strategy is rarely used because the porphyrin pyrrole units are inert towards most electrophilic reactions such as Friedel–Crafts alkylation and acylation, while halogenation^38^ and nitration^38^,^39^ often lead to di- or higher substituted products. An exception to this is the Vilsmeier–Haack reaction, which can provide mono-formyl porphyrins in appreciable yields.^39^ We reasoned that the resulting formyl group could subsequently be used to construct the desired D–s–A structure for the present studies. So, following Bonfantini's method,^39^ tetraphenylporphyrin (TPP, **1**) was converted to Cu(ii)–TPP and then subjected to the Vilsmeier–Haack reaction to obtain β-formyl-TPP (**2**). For the synthesis of the donor part of the molecule, *p*-nitrophenol was *o*-alkylated with either 1-bromohexene or 1-bromo-10-undecene to furnish compounds **3a** and **3b**, respectively. These were converted to aniline derivatives **4a** and **4b** by reduction with Zn/HCO_2_NH_4_. Next, aldehyde **2** was separately subjected to a reductive amination using **4a** or **4b** to obtain the target porphyrins **5a** and **5b**, respectively. Previously, Welch *et al.*^40^ synthesized the Schiff's base of **2** in toluene after 72 hr, using a Dean–Stark apparatus for simultaneous removal of water. We performed the reductive amination in THF in the presence of 4 Å molecular sieves followed by a one-pot reduction of the intermediate imine to obtain **5a** and **5b** in improved yields (∼78%) in only 6 h (Scheme 1).

### Device fabrication

#### Preparation of the Si-hybrids

**Fig. 1:**
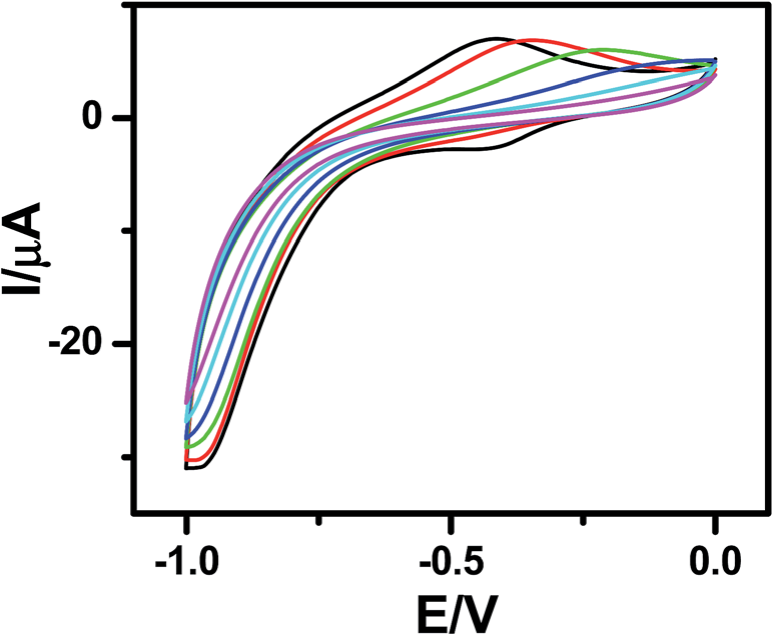
CVs indicating electrografting of **5b** molecules onto the Si (n++) wafers. The deposition was carried out using CV at a scan rate of 0.05 V s^–1^ under a N_2_ atmosphere with a Si wafer as the WE, Pt as the CE and Ag/AgCl as the RE. 0.1 M Bu_4_NP was used as the electrolyte and **5b** (1 μM) was used in dry CH_2_Cl_2_.

Molecules **5a** and **5b** were electrochemically deposited on H-terminated silicon *via* a two-step process, which is schematically shown in the ESI (Fig. SL1†). In the first step, application of a negative potential to the working electrode releases H free radicals from the Si–H surface. The newly generated nucleophilic Si atoms subsequently react with the alkene functionalities of **5a** and **5b** to form Si–C bonds, resulting in an irreversible oxidation peak at ∼0.3 V. A similar oxidation peak was observed with 1-undecene (Fig. SL2†), but not with the blank Si sample (electrolyte only), confirming our interpretation. Cyclic voltammograms (CVs) (Fig. 1), recorded during this electrochemical deposition helped to monitor the extent of deposition. Disappearance of the oxidation peak indicated completion of the process. AFM analysis revealed the formation of homogeneous monolayers with both **5a** and **5b** after 25 scans.

#### Characterization of the monolayers

**Fig. 2:**
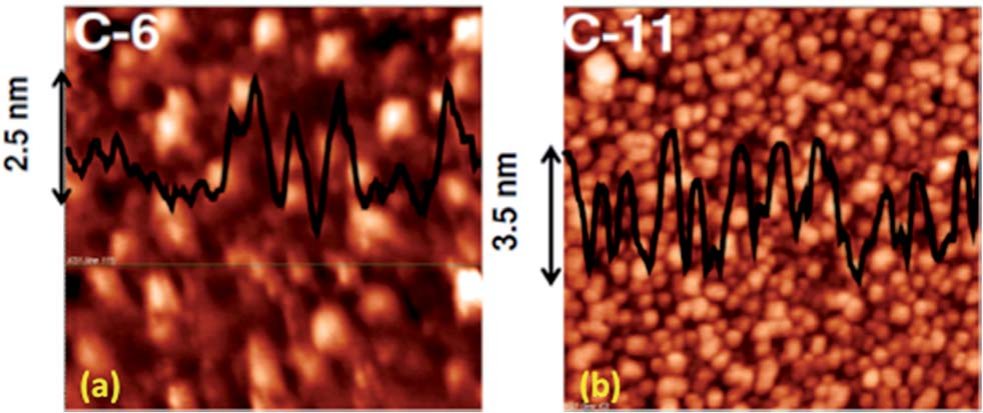
AFM images of (2 μm × 2 μm) (a) **5a** and (b) **5b** monolayers on Si (111).

To ensure monolayer deposition on Si, the electro-grafted materials were characterized by contact angle measurements, polarized FT-IR spectroscopy, ellipsometry, AFM, secondary ion mass spectrometry (SIMS) and electrochemistry. The contact angles of deionized water at the Si surface grafted with **5a** and **5b** were 55° and 64°, respectively. For the cleaned Si wafer and the C-11 alkyl-grafted Si surfaces, the angles were 84° and 112°, respectively. The value for the cleaned Si wafer is consistent with several previous reports.^41^–^45^ The low contact angles of the porphyrin monolayers suggested they were tilted on the Si-surface, exposing the pyrrole and amine nitrogen atoms for interaction with water droplets. The observed contact angles of the porphyrin monolayers are in close proximity to the reported contact values (66–74°) for thiophene-terminated alkyl monolayers on Si-surfaces that were prepared by a late-stage attachment of the aryl moieties.^46^ This established the suitability of our direct attachment protocol for the preparation of the monolayers. The average thicknesses of the monolayers, estimated by ellipsometry were found to be 2.4 ± 0.1 nm and 2.9 ± 0.2 nm in case of **5a** and **5b**, respectively. AFM analysis revealed that the monolayers formed after 25 scans were organized with the least number of voids and hillocks. The void depth and RMS roughness of the **5a** monolayers were ∼2.5 nm and 0.91 nm, respectively, while for **5b** they were 3 nm and 0.7 nm, respectively (Fig. 2). Compared to **5a**, the monolayers of **5b** were more compact and uniform with a larger grain size.

**Fig. 3:**
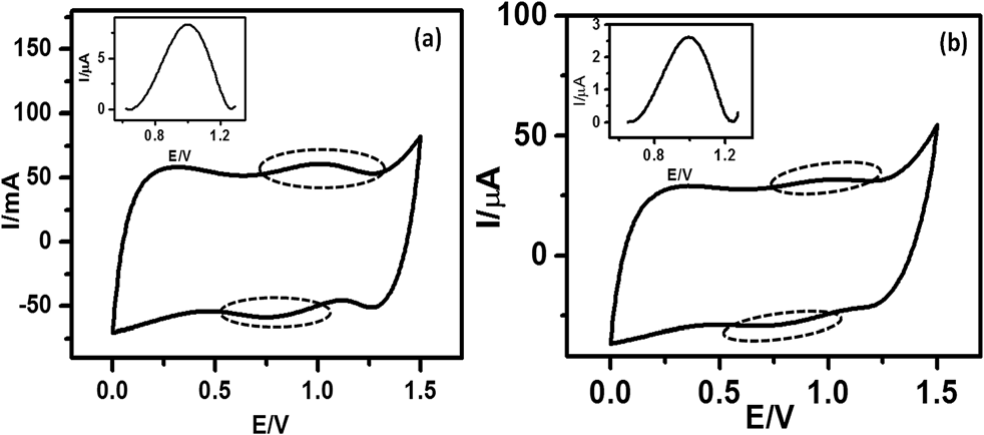
Fast scan CVs for the monolayers of (a) **5a** and (b) **5b** electro-grafted onto Si (n++) wafers. The CVs were recorded under a N_2_ atmosphere at a scan rate of 10 V s^–1^ using the respective monolayer-grafted Si as the WE, Pt as the CE, Ag/AgCl as the RE, and 0.1 M Bu_4_NP as the electrolyte. The dotted circles indicate the reversible peaks. The insets show the magnified redox peaks, after background corrections.

Fast scan (10 V s^–1^) CVs (Fig. 3) of the respective porphyrin monolayers exhibited a reversible peak at +0.8 V, confirming attachment of the porphyrin moieties. This was absent in the blank Si sample and the C-11 alkyl monolayers. The net charge transferred during the oxidation process, calculated from the area under the oxidation peak divided by the scan rate were 8.6 × 10^–7^ C and 2.45 × 10^–6^ C for **5a** and **5b**, respectively. Using these values, the surface coverages for the monolayers were calculated using the formula: surface coverage = total charge/(*F* × area dipped in electrolyte). The surface coverages were 1.11 × 10^12^ and 4.5 × 10^14^ molecules per cm^2^ for **5a** and **5b**, respectively. Thus, the areas occupied by each molecule in the **5a** and **5b** monolayers were 90 nm^2^ and 22 Å^2^, respectively, indicating that **5b** formed more compact monolayers than **5a**. The significantly higher value for **5a** compared to that previously reported^19^ for monolayers of simple C_18_-, C_16_-, and C_12_-alkanes on Si (100) revealed poor packing. This may be due to the edge-on orientation of the porphyrins.^47^ On the other hand, the value for the **5b** monolayers matched well with the theoretically calculated diameter (14.8 Å) of TPP,^48^ indicating that the molecules were tightly packed due to π–π stacking, which was also revealed in the AFM images (Fig. 2). Consistent with the AFM analysis, the surface area covered by **5b** was several fold that covered by **5a**.

**Fig. 4:**
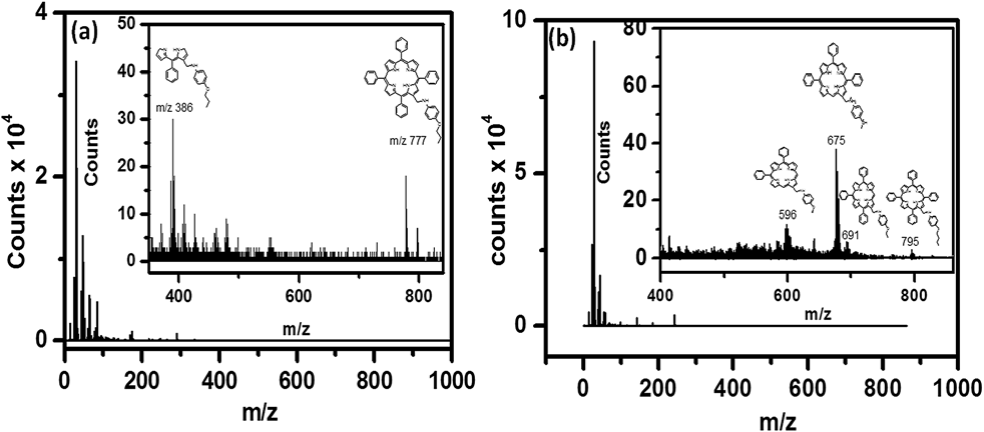
SIMS of the monolayers of (a) **5a** and (b) **5b** electro-grafted onto Si (n^++^) wafers.

SIMS of the **5a** monolayers showed mass peaks at *m*/*z* 795, 691, 675 and 596 amu, while for the **5b** monolayers peaks appeared at *m*/*z* 777 and 386 amu (Fig. 4), revealing that the molecules remained intact during the grafting process. The observed higher mass fragments, in the case of the C-11 monolayers, was consistent with its longer alkyl chain length *vis-à-vis* that of the C-6 monolayers.

**Fig. 5:**
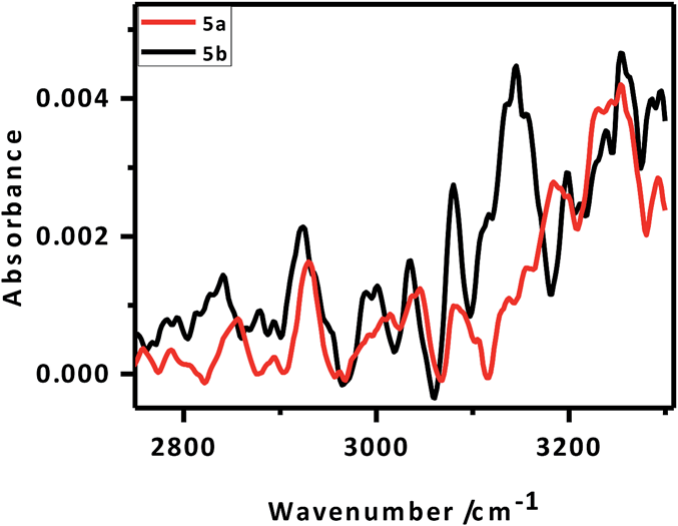
FTIR spectra of the monolayers of **5a** and **5b** electro-grafted onto Si (n^++^) wafers.

IR peaks due to –CH_2_ vibrational modes can provide better insight into the van der Waals interactions between the alkylated porphyrin rings anchored parallel on the Si surface. This, in turn, may help explain the better packing of the **5b** monolayers *vis-à-vis* that of **5a**. In pure solid alkane monolayers, the hydrocarbon chains exist in an all-*trans* configuration such that the carbon backbone of each molecule lies in a single plane. However, in liquid form, there is substantial out-of-plane twisting around the individual bonds, altering the frequency of the –CH_2_ vibrational modes.^45^,^49^ The polarized FTIR spectrum (Fig. 5) of the **5a** monolayers exhibited a N–H stretching frequency at 3251 cm^–1^ along with symmetric (*ν*_s_) and asymmetric stretching (*ν*_a_) vibrational modes for the CH_2_ groups at 2856 and 2927 cm^–1^, respectively. In contrast, the respective IR absorption peaks of the **5b** monolayers were at 3255, 2840 and 2921 cm^–1^. Our results showed that the alkyl chains in the monolayers of **5b** are more rigid like those in pure solid alkanes, while those in the monolayers of **5a** are twisted. This clearly explained the observed improvement in packing for the **5b** (C-11 linker) monolayers over the **5a** (C-6 linker) layers.^50^ From the X-ray photoelectron spectroscopy (XPS) data, the peak for the monolayers at 99.5 eV could be attributed to the Si–C bonds, while the absence of a SiO_2_ peak at 103 eV confirmed that the monolayers were free of SiO_2_ (Fig. SL3†).

#### *I–V* measurements

**Fig. 6:**
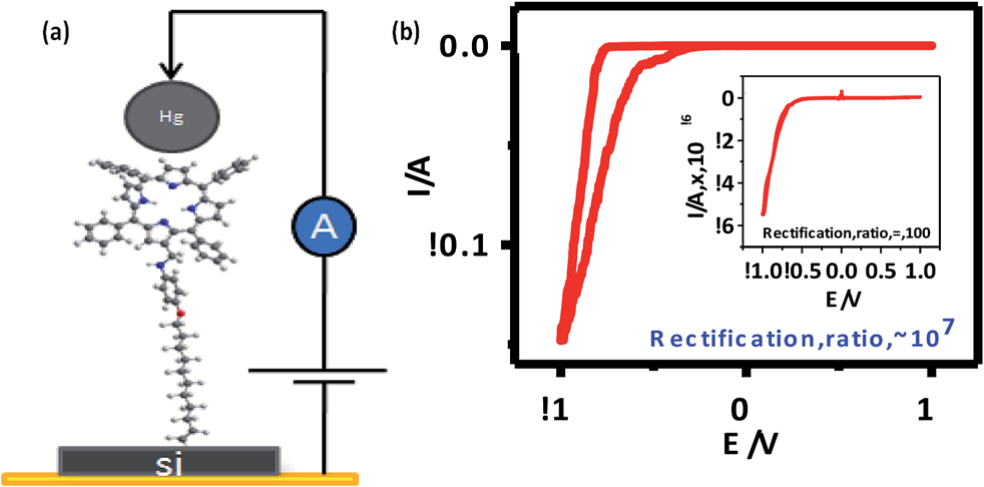
(a) *I–V* measurement set up; (b) experimental *I–V* characteristics of **5b** [inset: *I–V* curve for **5a**]. The *I–V* characteristics of all samples were similar, so representative curves are shown.

In order to measure the *I–V* characteristics, a metal–molecule–Si (n++) structure was constructed (Fig. 6(a)), using a tiny drop of liquid Hg (40 μm diameter) as the counter electrode. The area in contact with the grafted monolayer, measured using a goniometer, was 0.002 mm^2^.

The *I–V* curves (Fig. 6(b)) of the devices constructed using the monolayers of **5a** and **5b** showed current rectification in the reverse bias. The maximum RR was observed at ±1 V for both of the devices. The ratio for the monolayers prepared using **5b** was very high (10^7^), while that for **5a** (C-6 linker) was ∼100. However, while both systems were stable during repeated voltage scanning up to 100 scans at a scan rate of 0.01V s^–1^, the RRs reduced gradually from their original values to ∼10 000 for **5b** and ∼10 for **5a** after 50 scans.

It is already well known that when electrodes are asymmetric (and especially when the work functions of the electrode materials are different) any molecule can show current rectification.^51^,^52^ Therefore, to see the effect of using asymmetric electrodes (if any), we also recorded the *I–V* curves of two control devices made of (n++) Si/Hg (SL4 (a)†) and (n++) Si/C-11 alkyl monolayers/Hg (SL4 (b)†). These showed marginal rectifications with RR I^+^/I^–^ values of 0.75 and 2, respectively. Recently, we constructed a n^+^-Si/pyrene C-11 monolayers/Hg device, which exhibited current rectification in the positive bias with RR of 100 at 1 V.^53^ Furthermore, a n^+^-Si/5-(4-undecenyloxyphenyl)-10,15,20-triphenyl porphyrin (TPP C-11) monolayers/Hg system constructed by our group showed a marginally asymmetric *I–V* with significant hysteresis. In the positive bias scan (0 to +0.8 V), the current jumped by an order of magnitude at +0.6 V. However, on the reverse scan (+0.8 to 0 V) the current did not retrace the curve and remained at a higher value.^54^ Taken together, the *I–V* curves of all these devices clearly indicated that the results for the present devices were not due to electrode asymmetry. Moreover, the *I–V* results for the n^+^-Si/TPP C-11 monolayers/Hg device indicated that the observed rectifications in the negative bias using the **5a**/**5b** molecules were not due to resonance tunnelling through the TPP moiety.

The AFM and fast scan CV results showed better molecular stacking of the **5b** monolayers, which may be due to the longer alkyl bridge in **5b** than in **5a**.^55^ This may contribute to the better electrical characteristics of the **5b** monolayers because the overlapping of electron clouds favours the generation and transport of charge carriers to induce intrinsic conductivity. Consequently, a significantly higher maximum RR was observed for the **5b** monolayers. Control experiments, carried out with a blank Si sample as well as C-11 alkyl chain-grafted Si-wafers showed nearly symmetrical sigmoidal *I–V* curves (Fig. SL4†), eliminating any doubt about artifacts.

**Fig. 7:**
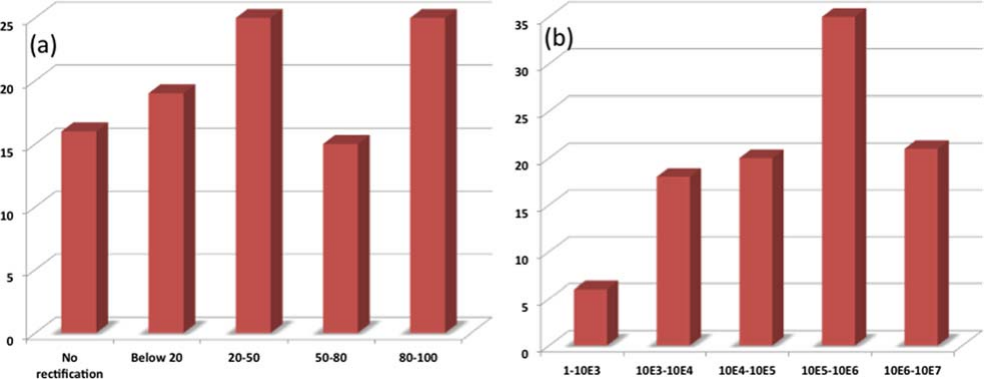
Statistics for the RR values for the devices made with (a) **5a** and (b) **5b**.

The void sizes (∼0.2–0.4 nm) of the present Si–alkyl porphyrin/Hg junctions were small compared to the size of the Hg drops (∼40 μm). Therefore, Hg drops are unable to penetrate through the pinholes of the SAMs and the measured *I–V* is expected to be direct. Statistical analyses of the data and junction yields are extremely valuable to discriminate artifacts from the real data. Previously, Kim *et al.*,^56^ and Nijhuis *et al.*,^57^,^58^ employed extensive statistical analyses to assess the performance of SAM-based devices. In the present work, we constructed only 80 devices for each of compounds **5a** and **5b**. Nevertheless, we analyzed the statistics of our *I–V* results as shown in Fig. 7 and Table 1, and summarized below. For compound **5a**, only 25% of the devices showed RR values of 80–100, while an additional 15% of the devices showed RR values of 50–80. However, the RR values of 44% of the devices were <50, while 16% of the devices didn't show any rectification. The device statistics for the monolayers of compound **5b** were very impressive. The RR values for the majority (35%) of the devices were 10^5^–10^6^, while 21% of the devices showed RR values of 10^6^–10^7^. Another 20% showed RR values of 10^4^–10^5^, and the rest had RR values of 1–10^4^. The performance of the devices made of compounds **5a** and **5b** were satisfactory. In particular, the RR values of the Hg/**5b**/Si (n++) devices were far superior to that of molecular rectifiers reported so far.^53^,^57^,^58^

**Table 1:** Statistics for the *I–V* data

Molecule	No. of samples	No. of devices in each sample	Total no. of devices	No. of devices exhibiting rectification
**5a**	10	8	80	67
**5b**	10	8	80	80

The current rectification properties of various D–s–A-based SAMs and LB films in contact with noble metal electrodes have been described.^59^ Results for some representative examples clearly establishes the significantly superior performance of the devices described in the present study. For example, a LB film of a pyrenyl carbamate in a M–M–M junction exhibited a RR value of 130 at ∼2.5 V.^60^ Likewise, quinolinium and tetrahydroisoquinolinium iodide-based SAMs deposited on Au substrates showed RR values of 50–150 and 30–80, respectively, at ±1 V.^61^,^62^ Meanwhile, the RR value for SAMs of quinolinium salts joined by a truncated S–C_3_H_6_ group on a Au surface was found to be 12 at ±1 V.^63^ In another study, a LB monolayer of the D^+^–π–A^–^ molecule, hexadecylquinolinium tricyanoquinodimethanide on Au electrodes showed a maximum RR of 27.5 at 2.2 V.^64^

However, literature reports on metal–porphyrin–semiconductor junctions are scarce. SAMs of 4-aminothiophenol/ZnTPP/fulleropyrrolidine (PyC_2_C_60_) on a Au (111) surface showed a modest RR of 24 at 1.8 V.^35^ Interestingly, self-assembled layers of Fe(iii)-5,15-di[4-(*s*-acetylthio)phenyl]-10,20-diphenyl porphyrin on annealed Au crystal facets on glass substrates showed asymmetric *I–V* curves with the highest RR up to 9000, but the majority of the devices showed RR = 20–200 at ±1 V.^65^

To confirm our current rectification results, we computed the theoretical *I–V* curve of the device made of **5b**. Initially, the ground state (GS) geometry of molecule **5b** was optimized using an *ab initio* molecular orbital theory based LCAO-MO approach as implemented in the GAMESS software. The ionic optimization of molecule **5b** was carried out without any symmetry constraint at the B3LYP/6-31G(d,p) level of theory. To calculate the transport characteristics, a suitable device was constructed using the optimized configuration of the molecule as the central device region between two electrodes. Besides the active parts of the device, the central region also included a sufficient part of the contacts, such that the properties of the electrode regions could be described as bulk materials. This could be ensured by extending the central region into a few layers of the metallic contacts. The calculation of the electron-transport properties of the system was divided into two parts: (i) a self-consistent calculation for the electrodes with periodic boundary conditions in the transport direction, and (ii) a self-consistent open boundary calculation of the properties of the central region, where the electrodes define the boundary conditions. The complete details of the method are described in the literature.^66^

**Fig. 8:**
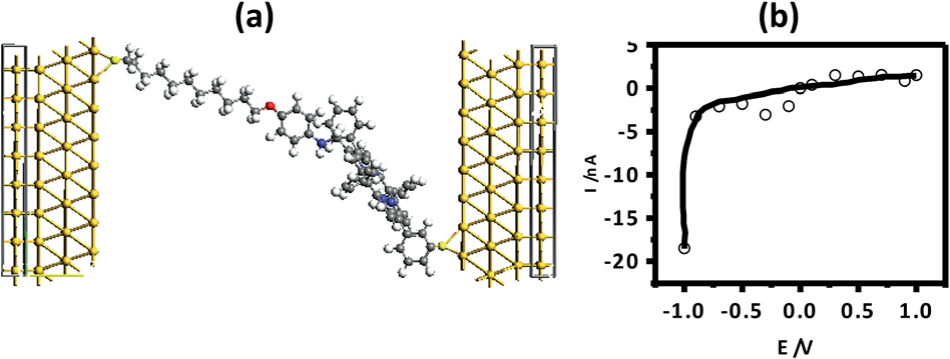
(a) Theoretical device geometry for Au/molecule **5b**/Au. (b) Theoretical *I*–*V* curve.

In the present experimental set-up, we have used a highly doped Si substrate that is expected to undergo reconstruction. This restricted accurate modelling of the molecule–substrate interface using the *ab initio* formalisms of our computational resources. Therefore, a model for a two-probe system was constructed (Fig. 8(a)) by placing the molecules between two Au electrodes. We modelled the electrodes as part of truncated solid crystals. Unlike Hg, which is liquid under the experimental conditions and has a complicated structure, Au possesses a well-defined face-centred cubic crystal structure. Additionally, the pseudo-potential for Au is robust and it has been tested and used by many groups as a model electrode.^67^,^68^ Understandably, the chosen system is not ideal for verification of the experimental results. However, our calculations were primarily aimed at a qualitative understanding of the electron transport through these molecules, and not a quantitative comparison, justifying our choice of Au electrodes. It is worth noting that Zheng *et al.*^69^ recently reported the NDR properties of C_60_ based electronic devices, wherein they claimed that the findings were independent of the type of electrodes used. For construction of the theoretical device, a thiol end group was used for attachment of the molecule with the electrode. The interface geometry of the thiol-terminated molecule and the electrode was optimized to ensure good overlap between the device and the electrodes.

Previously, we have reported the interaction of methyl thiol, a prototype device molecule, with an extended Au(111) surface using a plane wave based pseudopotential method.^70^ The results showed that the terminal S atom binds at the hollow site of the Au(111) surface and the distance between the Au and S atoms is 2.52 Å. Using this information, we constructed the model for our present calculations. Two Au(111)-8X8 surfaces were used as the left and right electrodes. The Au/molecule/Au configuration was divided into three parts: left electrode, right electrode, and the central scattering region. In our models, there were three Au layers in each of the left and right electrode unit cells. The scattering region was composed of the isolated molecule together with the respective two Au layers on the left and right sides. The electron-transport properties of the Au/molecule/Au systems were investigated using the ATK 11.2.3 program, where semi-empirical extended Hückel theory, in combination with a first-principle NEGF, was employed.^71^ A *k*-point sampling of 100 was used in the electron-transport direction (*Z* direction).^70^ Consistent with the experimental results, the theoretical *I–V* curve also showed rectification in the reverse bias (Fig. 8(b)).

In general, the forward bias current-flow should be determined by the HOMO states of the molecules, while their respective LUMO states would dictate the reverse bias current. The observed rectification in the reverse bias is a result of an alignment of the LUMO levels of the molecules with the Fermi-levels of the electrodes. To verify this, we determined the HOMO and LUMO energy levels (Table 2) of **5b** by theoretical calculations using an *ab initio* method (GAMESS software). Ionic optimization without any symmetry constraints was carried out at the B3LYP/6-31G(d,p) level of theory where the exchange correlation functions are expressed using hybrid density functional theory. It was observed that the HOMO of the molecule was at –4.707 eV and the LUMO at –2.062 eV. The HOMO of the molecule (located at *p*-aminophenol group) is in close proximity to the Fermi level of electrode, but due to non contact with the electrode, resonance tunnelling will be difficult. For the LUMO, the energy difference with the electrode Fermi level is too large to undergo resonance tunnelling. Therefore, the observed current rectification is unlikely to be due to resonant tunnelling, but molecular asymmetry (D–s–A), which favours Aviram and Ratner's mechanism for rectification.

**Table 2:** Energy levels for tetraphenylporphyrin, *p*-aminophenol and **5b**

Energy values in eV			
	Tetraphenylporphyrin	*p*-Aminophenol	**5b**
HOMO–2	–6.53074	–7.97294	–5.4757
HOMO–1	–5.52391	–7.04775	–5.1448
HOMO	–5.30622	–5.41507	–4.7990
LUMO	–2.55787	–0.48981	–2.1984
LUMO+1	–2.53066	–0.05442	–2.1739
LUMO+2	–0.9524	0	–0.5831

**Fig. 9:**
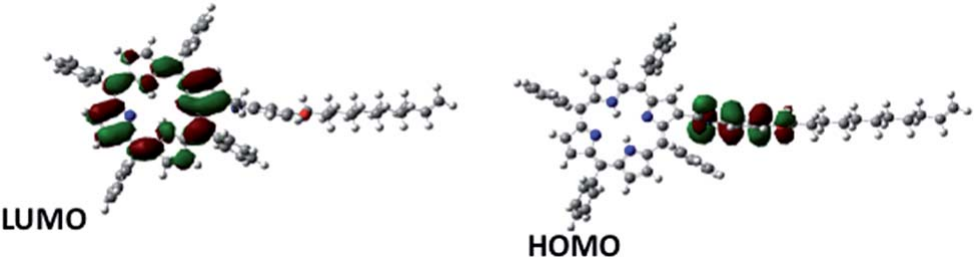
Spatial distribution of the HOMO and LUMO energy levels of **5b**.

From the spatial distribution of the HOMO and LUMO energy levels (Fig. 9), it could be seen that the HOMO was localized on the *p*-aminophenol segment, while the LUMO was on the porphyrin ring, and their separation by the spacer, CH_2_, allowed a unidirectional flow of electrons. This is consistent with the Aviram and Ratner theory that suggests that a single molecule with a donor–spacer–acceptor (D–s–A) structure should behave as a diode when placed between two electrodes if a non-conjugated σ-bond bridge prevents direct overlap of the donor and acceptor energy levels. Moreover, a monolayer will rectify if its molecules are aligned in register between two electrodes such that they work together when electrons flow from the electrode MD (attached to the acceptor) to D, and then exit from A to the electrode MA (attached to the donor).

**Fig. 10:**
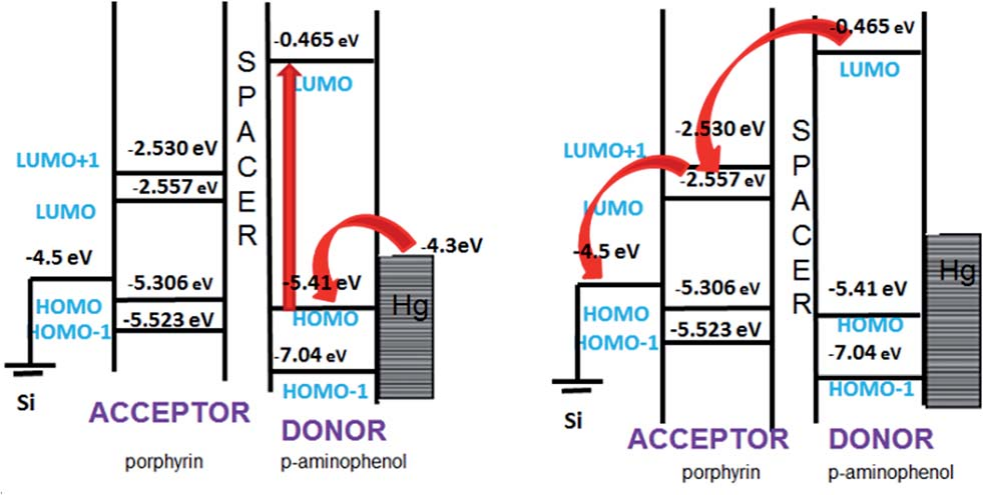
Mechanism of rectification for the devices made with **5b**.

To underscore the mechanism of electron flow between the donor and acceptor moieties, we also calculated the energy levels of the individual components (*p*-aminophenol and TPP) of **5b** using the same computational approach. The energy level diagram of the constituent species is shown in Fig. 10. The LUMO energy level of *p*-aminophenol was at a higher energy than the TPP moiety. This will make it the donor. Therefore, under reverse bias, when electrons flow from MD to D, the electrons will move from the HOMO of *p*-aminophenol to its LUMO, tunnel through the bridge to the vacant LUMO of TPP, and finally transfer to the Hg electrode to complete the reverse-direction flow.

In principle, reversing the orientation of the molecular dipole would change the *I–V* curves of the devices. This would also confirm that the observed rectification is not simply because of the non-symmetric nature of the device system.^72^ However, construction of this type of device is synthetically more demanding for the following reasons. The synthesis has to start with an unsymmetrical porphyrin, wherein the porphyrin needs to have the alkenyl attachment at one of its pyrrole moieties for grafting to the Si wafers. Synthesis of the required porphyrin proceeds *via* mono-bromination of the porphyrin followed by a Stille coupling with 11-trimethylstannyl undecene. Next, the unsymmetrical porphyrin needs to be formylated for the subsequent attachment of the aniline moiety. However, formylation of unsymmetrical porphyrins is never clean, and we experienced the formation of a mixture of products, formylated at different sites, and their purification was extremely cumbersome. Moreover, the grafting of such a molecule may not self-assemble in a similar manner to that used in the present work. Thus, the *I–V* results of the newly constructed device may provide unreliable results, despite having the reverse geometry. To confirm this hypothesis, the GS geometry of a **5b**-congener having the alkenyl attachment at one of the pyrrole moieties in the porphyrin core was theoretically optimized, as done for **5b**. The GS geometry of the congener revealed that both the porphyrin and aniline moieties lie on same side (Fig. SL5†). Therefore, the Si-grafted **5b**-congener would be positioned in such a manner that the Hg electrode would preferably interact with the porphyrin core. However, **5b** has many possible conformers, and the possibility of touching the mercury electrode by one of its straight conformers cannot be excluded. Nevertheless, based on the *I–V* results of the TPP C-11 monolayers^54^ and that of **5a**/**5b**, it is tempting to propose that the observed current rectification is because of the nature of the molecules.

## Experimental section

### General

#### Experimental details

Synthesis: all reagents and solvents (Sigma-Aldrich and Fluka) were of synthetic grade. Propionic acid, pyrrole, benzaldehyde, *p*-nitrophenol were used after recrystallisation. All solvents were dried and distilled before use. Tetrahydrofuran (THF) and hexane were distilled over Na under argon. DMF was dried with CaH_2_ and distilled under vacuum. The ^1^H NMR and ^13^C NMR spectra were recorded with 200/300/500 (50/75/100) MHz spectrometers using deuterated solvents as internal standards. The mass spectrometry was carried out with an MS/MS (410 Prostar Binary LC with 500 MS IT PDA Detectors, Varian Inc, USA) and MALDI-TOF/TOF (Bruker Ultraflex II) data systems. The IR spectra were recorded as films with a Jasco model A-202 FT-IR spectrometer and only the pertinent bands are expressed.

### Synthesis

#### 5,10,15,20-Tetraphenylporphyrin (**1**)

To a refluxing solution of benzaldehyde (5.30 g, 50 mmol) in propanoic acid was added pyrrole (3.35 g, 50 mmol) in propanoic acid drop wise. After refluxing for 2 h under stirring, the mixture was cooled to room temperature and left for 12 h. The mixture was filtered, then the precipitate washed with methanol, dried in vacuum, and subjected to column chromatography (silica gel, 50% CHCl_3_/hexane) to obtain **1** (1.62 g, 20%) after crystallization. Purple crystals; m.p.: >250 °C (MeOH/CHCl_3_); *δ*_H_(200 MHz; CDCl_3_; Me_4_Si) –2.78 (broad s, 2H), 8.84 (s, 8H), 8.21 (m, 8H), 7.76 (m, 12H); MALDI-TOF (HCCA matrix): *m*/*z* (%) 614 d.

#### 5,10,15,20-Tetraphenylporphyrin-2-carbaldehyde (**2**)^38^

To a refluxing and stirred solution of **1** (0.500 g, 0.814 mmol) in CHCl_3_ (75 mL) was added Cu(OAc)_2_·H_2_O (0.179 g, 0.895 mmol) in MeOH (12 mL). On completion of the reaction (*cf.* TLC, 30 min) the reaction mixture was brought to room temperature and triturated with MeOH to obtain the corresponding Cu(ii)–porphyrinato acetate (0.647 g, ∼quant.) as a purple powder. m.p.: >250 °C (MeOH/CHCl_3_); UV-Vis (CH_2_Cl_2_) *λ*_max_ [nm]: 302, 416, 540, 576, 617; MALDI-TOF: *m*/*z* (%): 795 d.

POCl_3_ (7.9 mL, 52 mmol) was added drop wise to anhydrous DMF (5.5 mL, 75.6 mmol) at 0 °C to obtain the Vilsmeier complex as a thick golden liquid. To this was added a cold suspension of the Cu(ii)–porphyrinato salt (0.500 g, 0.73 mmol) in dichloroethane (50 mL). The reaction mixture was brought to room temperature, refluxed for 5 h, cooled to room temperature, and left overnight. Concentrated H_2_SO_4_ (10 mL) was added to the ice-cold mixture and stirring continued for 10 min. The green mixture was poured into ice-cold aqueous NaOH (0.625 M, 1 L) with occasional shaking until disappearance of the green colour. The mixture was extracted with CHCl_3_ (2 × 200 mL), then the organic layer was washed with saturated NaHCO_3_ (2 × 350 mL) until neutral before being dried over MgSO_4_. Solvent removal *in vacuo* followed by column chromatography (silica gel, 40% CHCl_3_/hexane) of the residue gave **2** (0.085 g, 75%). m.p.: >250 °C (MeOH/CHCl_3_); UV-Vis (CH_2_Cl_2_) *λ*_max_ [nm]: 431, 526, 567, 606, 664; *δ*_H_ (600 MHz; CDCl_3_; Me_4_Si) 9.40 (m, 1H), 9.24 (s, 1H), 8.90 (m, 4H), 8.78 (d, *J* = 2.3 Hz, 2H), 8.25 (d, *J* = 7.0 Hz, 2H), 8.20 (m, 6H), 7.79 (m, 12H); *δ*_C_ (75 MHz; CDCl_3_; Me_4_Si) 189.4, 142.5, 141.9, 141.7, 135.1, 134.7, 133.5, 130.8, 129.1, 128.5, 128.2, 128.0, 127.7, 127.5, 127.1, 126.9, 122.7, 120.7, 120.4, 120.1; MALDI-TOF: *m*/*z* (%) 642 d; LCMS *m*/*z* (%): 643.3 amu. Found: C 83.84; H, 4.82; N, 8.49%. Calcd for C_45_H_30_N_4_O: C 84.09; H, 4.70; N, 8.72%.

#### 4-(5-Hexenyloxy)-1-nitrobenzene **3a** and 4-(10-undecenyloxy)-1-nitrobenzene **3b**

A stirred mixture of *p*-nitrophenol (0.500 g, 3.59 mmol), 10-undecenyl bromide (0.920 g, 3.95 mmol) or 5-hexenyl bromide (0.645 g 3.95 mmol) and K_2_CO_3_ (0.644 g, 5.14 mmol) in dry acetone was refluxed for 12 h. The reaction mixture was cooled, filtered over celite, concentrated and residue dissolved in CHCl_3_ (20 mL). The organic phase was washed with H_2_O (2 × 20 mL) and brine (1 × 5 mL), and then dried. Removal of the solvent followed by column chromatography (silica gel, 2% EtOAc/hexane) of the residue furnished **3a** (0.791 g, 99%) and **3b** (1.0 g, 99%) as gels.

**3a**: *δ*_H_ (600 MHz; CDCl_3_; Me_4_Si) 8.18 (m, 2H), 6.93 (m, 2H), 5.83 (m, 1H), 5.01 (m, 2H), 4.06 (t, *J* = 6.0 Hz, 2H), 2.14 (q, *J* = 6.0 Hz, 2H), 1.84 (quint, *J* = 6.8 Hz, 2H), 1.58 (m, 2H); *δ*_C_ (75 MHz; CDCl_3_; Me_4_Si) 164.2, 141.3, 138.3, 125.9, 115.0, 114.4, 68.7, 33.3, 28.4, 25.2; LCMS *m*/*z* (%): 222.0 amu. Found: C, 65.26; H, 6.77; N, 6.33%. Calcd for C_12_H_15_NO_3_: C, 65.14; H, 6.83; N, 6.33%.

**3b** *δ*_H_ (600 MHz; CDCl_3_; Me_4_Si) 8.18 (m, 2H), 6.93 (m, 2H), 5.94 (m, 1H), 4.93 (m, 2H), 4.04 (t, *J* = 6.0 Hz, 2H), 2.04 (q, *J* = 6.0 Hz, 2H), 1.82 (quint, *J* = 6.6 Hz, 2H), 1.28 (m, 14H); *δ*_C_ (75 MHz; CDCl_3_; Me_4_Si) 164.3, 141.3, 139.2, 125.9, 114.4, 114.2, 68.9, 33.8, 29.5, 29.4, 29.3, 29.1, 29.0, 25.9; LCMS *m*/*z* (%): 292.0 amu. Found: C, 70.47; H, 8.78; N, 4.59%. Calcd for C_17_H_25_NO_3_: C, 70.07; H, 8.65; N, 4.81%.

#### 4-Hexenyloxyaniline **4a** and 4-undecenyloxyaniline **4b**

To a stirred mixture of **3a** or **3b** (0.684 mmol) and HCO_2_NH_4_ (0.068 g) in MeOH (5 mL), was added Zn dust (0.054 g, 0.82 mmol) under Ar. After 10 min, the mixture was filtered through celite and washed with Et_2_O (2 × 20 mL). The organic layer was washed with H_2_O (2 × 15 mL) and brine (1 × 5 mL), and then dried. Removal of the solvent *in vacuo* afforded **4a** (0.116 g, 89%) and **4b** (0.162 g, 91%) as white powders. The samples turned brown very fast, so were used for the next step without further purification.

#### Porphyrins **5a** and **5b**^39^

A mixture of **2** (0.075 g, 0.12 mmol), **4a** or **4b** (0.17 mmol), 4 Å molecular sieves (0.030 g) and AcOH (2 drops) in THF (5 mL) was refluxed until consumption of the starting materials (*cf.* 3 h). NaBH_3_CN (0.008 g, 0.15 mmol) in MeOH (5 mL) was added into the respective mixtures, which were then refluxed for an additional 3 h. The mixtures were diluted with H_2_O (5 mL) and extracted with CHCl_3_ (50 mL). The organic layers were washed with H_2_O (2 × 50 mL) and brine (1 × 5 mL), and then dried. Removal of solvent *in vacuo* afforded residues, which upon purification with column chromatography (silica gel, 40% EtOAc/hexane) gave **5a** (0.075 g, 78%) as a gel and **5b** (0.080 g, 78%) as a solid.

**5a**: UV-Vis (CH_2_Cl_2_) *λ*_max_ [nm]: 410, 514, 549, 589, 645; *δ*_H_ (300 MHz; CDCl_3_; Me_4_Si) 8.82 (m, 6H), 8.64 (d, *J* = 4.8 Hz, 1H), 8.17 (m, 8H), 7.73 (m, 12H), 6.72 (m, 2H), 6.48 (m, 2H), 5.83 (m, 1H), 4.98 (m, 2H), 4.45 (s, 2H), 3.88 (t, *J* = 6.6 Hz, 2H), 1.97–2.19 (m, 3H), 1.76 (m, 2H), 1.54 (dt, *J* = 15.3, 7.6 Hz, 2H), –2.79 (broad s, 2H); *δ*_C_ (75 MHz; CDCl_3_; Me_4_Si) 151.7, 142.4, 142.3, 142.1, 141.9, 138.7, 134.7, 134.6, 133.2, 128.5, 127.8, 127.7, 127.3, 126.8, 126.7, 120.7, 120.3, 119.5, 119.4, 115.7, 114.7, 68.6, 45.1, 33.6, 29.8, 29.0, 25.4; MALDI-TOF: *m*/*z* (%): 818. Found: C, 83.04; H, 6.08; N, 8.04%. Calcd for C_57_H_47_N_5_O: C, 83.69; H, 5.79; N, 8.56%.

**5b**: m.p.: >250 °C; UV-Vis (CH_2_Cl_2_) *λ*_max_ [nm]: 411, 514, 548, 589, 646; *δ*_H_ (600 MHz; CDCl_3_; Me_4_Si) 8.70 (m, 6H), 8.64 (d, *J* = 5.9 Hz, 1H), 8.22 (m, 4H), 8.12 (m, 4H), 7.73 (m, 12H), 6.70 (m, 2H), 6.48 (d, *J* = 8.2 Hz, 2H), 5.81 (dd, *J* = 17.0, 10.0 Hz, 1H), 5.01 (d, *J* = 16.4 Hz, 1H), 4.95 (d, *J* = 10.6 Hz, 1H), 4.45 (broad s, 2H), 3.87 (t, *J* = 7.0 Hz, 2H), 3.74 (broad s, 1H), 2.11 (m, 2H), 1.74 (m, 2H), 1.54 (t, *J* = 8.2 Hz, 2H), 1.20–1.31 (m, 10H), –2.77 (broad s, 2H); *δ*_C_ (75 MHz; CDCl_3_; Me_4_Si) 151.8, 142.3, 142.2, 142.1, 141.9, 139.3, 134.7, 134.6, 133.2, 128.5, 127.8, 127.7, 127.3, 126.8, 126.7, 117.4, 115.7, 115.6, 114.7, 114.2, 68.8, 68.7, 33.9, 32.0, 29.8, 29.7, 29.6, 29.5, 29.2, 29.0, 26.1, 22.8, 22.2; MALDI-TOF *m*/*z* (%): 888. Found: C, 83.40; H, 6.17; N, 8.63%. Calcd for C_62_H_57_N_5_O: C, 83.84; H, 6.47; N, 8.56%.

#### Characterization of the monolayers

The monolayers were characterized in terms of thickness, using an ellipsometer (Sentech, model SE 400adv); surface morphology was measured by AFM imaging (Nanonics, Multiview 4000 system), de-ionized water contact angle (Data Physics System, model OCA20), FT-IR (Bruker, 3000 Hyperion Microscope with Vertex 80 FTIR System, LN-MCT 315-025 detector) in polarized ATR mode (20× objective) at an angle of 45° for 500 scans and the data were background corrected with freshly prepared Si–H monolayers. The molecular mass was measured by SIMS (BARC make, Kore's Technology software) keeping Si–H as a reference. The XPS analysis of the deposited films was carried out using a Mg Kα (1253.6 eV) source and a MAC-2 electron analyzer. The XPS analysis chamber was maintained at a base vacuum of 10^–9^ mbar. The XPS binding energy scale was calibrated to the Au 4f_7/2_ line at 83.95 eV.

#### Preparation of H-terminated Si wafers

n-Type silicon wafers (orientation: 111; resistivity: 0.001–0.005 Ω cm) and 40% NH_4_F were purchased from Siltronix and Fluka, respectively. The Si (111) wafers were cut into small pieces (∼0.5 cm × 1.5 cm) and cleaned by heating in a 3 : 1 (v/v) mixture of conc. H_2_SO_4_ : 30% H_2_O_2_ (piranha solution) for 10 min at 80 °C. The wafers were washed with excess H_2_O and immersed successively in deaerated (purged with Ar for 30 min) 40% aqueous NH_4_F for 10 min and 2% aqueous HF for 2 min. The wafers were washed with deionized H_2_O for 1 min, dried under a stream of N_2_ and immediately taken into the electrochemical cell for electro-grafting.

#### Monolayer formation

The electrochemical deposition of **5a** and **5b** was carried out by cyclic voltammetry (CV) with a potentiostat/galvanostat system (model: Autolab PGSTAT 30) using Si wafers as the working electrode (WE), Pt as the counter electrode (CE) and Ag/AgCl as the reference electrode (RE). The solution contained 0.1 M Bu_4_NP as the electrolyte and **5a** or **5b** (1 μM) in dry CH_2_Cl_2_. The CV was run from 0 to –1 V for 30 cycles at a 0.05 V s^–1^ scan rate under an inert atmosphere. After the CV scans, the WE was sonicated in CH_2_Cl_2_ for 10 min to remove the electrolyte and any unreacted or physisorbed **5a** or **5b**. The WE was further washed with acetone, isopropanol and methanol to obtain the respective grafted monolayers.

#### Junction and measurement setup

To measure the *I*–*V* characteristics, a metal/molecule/Si (n++) structure was completed by using a tiny drop of liquid mercury of diameter 40 ± 2 μm as the counter electrode. The contact area in the grafted monolayer was 0.002 mm^2^. The *I*–*V* curves were recorded at room temperature in a dark box using a pA meter-dc voltage source (HP 4140).

#### Theoretical calculations

The ground state geometry optimization and molecular orbital calculations of molecule **5b**, TPP, *p*-aminophenol and the **5b** congener were carried out using an *ab initio* molecular orbital theory based LCAO-MO approach as implemented in the GAMESS software. The ionic optimization of the molecules was carried out without any symmetry constraints at the B3LYP/6-31G(d,p) level of theory.

## Conclusions

Overall, we have synthesized two porphyrin-based D–s–A prototype systems (**5a** and **5b**) with two alkenylated anilines, differing in the chain length of the alkenyl using C-6 and C-11, respectively. These were individually electro-grafted on H-terminated Si surfaces to form monolayers. The *I*–*V* characteristics of the monolayers revealed pronounced, stable and reversible current rectification at room temperature in the negative bias. To the best of our knowledge, such high RR values are rare, except for previous devices constructed by C. A. Nijhuis and Whitesides's group,^51^,^57^,^58^ L. Venkataraman^52^ and a recent publication from our own group.^53^ The monolayer with the C-11 linker was more compact and showed a 10^5^ times high rectification ratio (RR) relative to the other similar system having the C-6 linker, possibly because of the compact packing. The rectification mechanism was explained on the basis of Aviram and Ratner’s theory of rectification by using *ab initio* molecular orbital calculations.

## Supplementary Material

Supplementary information
