# Supplementary material for: A novel design for porphyrin based D–s–A systems as molecular rectifiers
Source: Chem Sci. 2015 Nov 16;7(2):1548–57. doi: 10.1039/c5sc03590b (PMC5964962; doi:10.1039/c5sc03590b)
Supplement: Supplementary file 1 [file SC-007-C5SC03590B-s001.pdf]

Electronic Supplementary Material (ESI) for Chemical Science.
This journal is © The Royal Society of Chemistry 2015

Supplementary material

Novel design of porphyrin based D-s-A systems as molecular rectifiers

Kavita Garg,[a*] Chiranjib Majumder,[b] Dinesh Kumar Aswal,[c] Sandip Kumar Nayak,[a] and Subrata Chattopadhyay[a]

[a]Bio-Organic Division, [b]Chemistry Division, [c] Technical Physics Division,

Bhabha Atomic Research Centre, Mumbai – 400085, India.

E-mail: kavitachemistry1@gmail.com, dkaswal@yahoo.com

Content

s.no	Title	Page no.
1	**Figure. SL1.** Mechanism of electrochemical deposition	3
2	**Figure SL2.** Cyclic voltammograms (CVs) of C-11 alkylmonolayers indicating electrografting of the molecules on silicon (n^{++}) wafers.	3
3	**Figure SL3.** XPS of monolayers of **6b**	
4	**Figure SL4.** Experimental *I-V* characteristics of devices (a) Hg/ undeposited Si(n^{++}). (b) Hg/ C-11alkyl monolayers/Si(n^{++})	3
5	**Figure SL5**. The optimized geometry of the 5b-congener that has alkenyl group at the porphyrin ring.	4
6	Input file for p-aminophenol	5
7	Input file for tetraphenylporphyrin	6
8	Input file for **5b**	8
9	Input file for **5b**-congener that has alkenyl group at the porphyrin ring	11

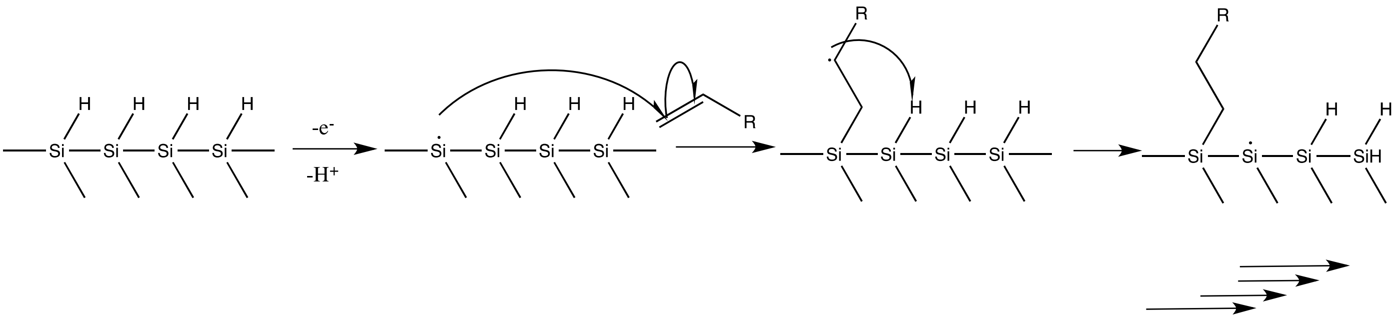

Figure. SL1. Monolayer deposition mechanism

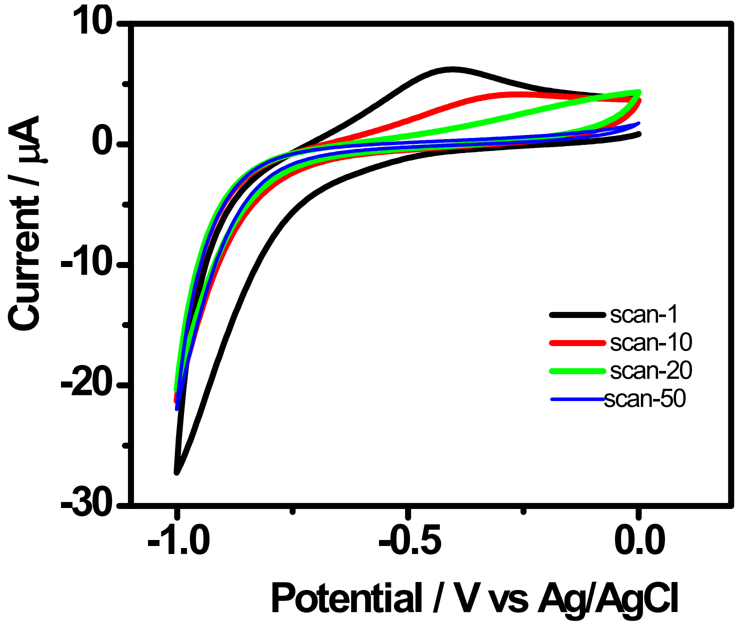

Figure SL-2 Cyclic voltammograms (CVs) of C-11 alkylmonolayers indicating electrografting of the molecules on silicon (n^{++}) wafers.

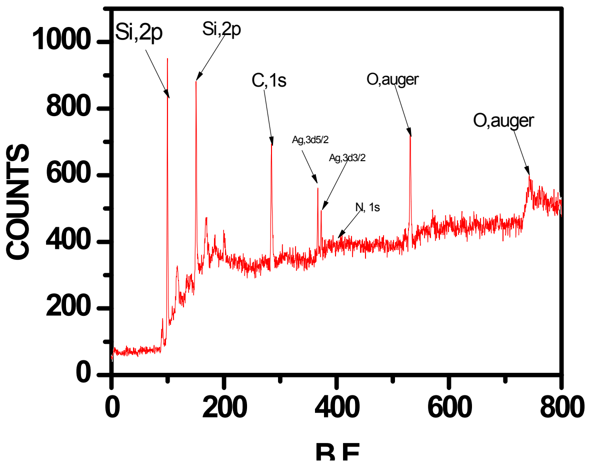

Figure SL3. XPS of monolayers of **6b**

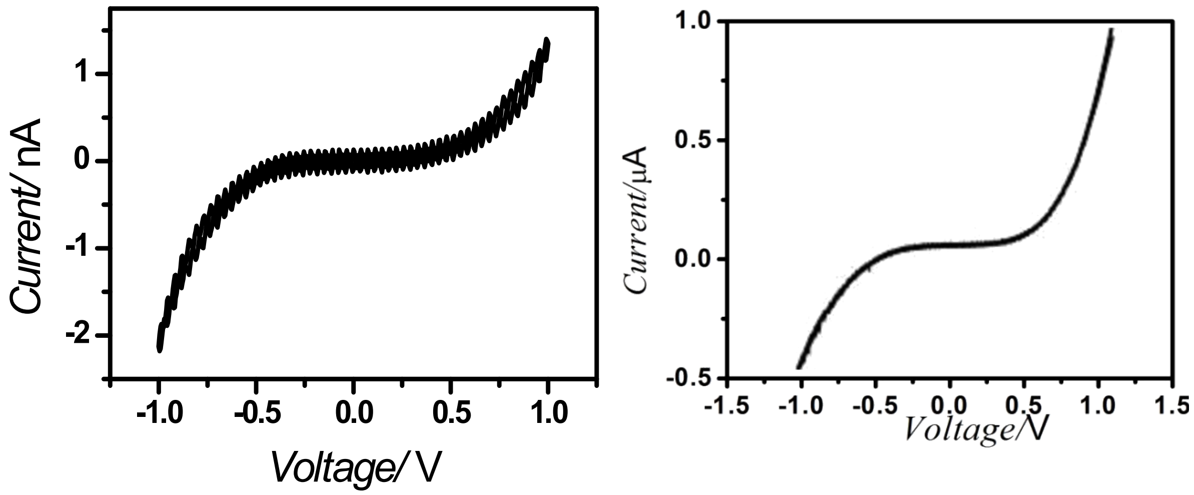

Figure SL-4. Experimental *I-V* characteristics of devices (a) Hg/ undeposited Si(n^{++}). (b) Hg/ C-11alkyl monolayers/Si(n^{++})

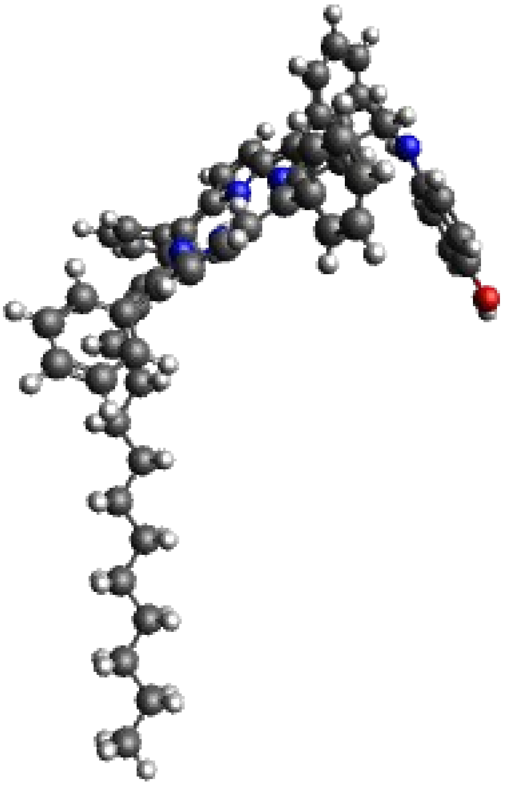

Figure SL-5. The optimized geometry of the **5b**-congener that has alkenyl group at the porphyrin ring.

Input file for p-aminophenol

```
$SYSTEM TIMLIM=600000.0 MEMORY=51200000 $END
 $CONTRL SCFTYP=RHF MPLEVL=0 MAXIT=200 ICHARG=0 MULT=1 COORD=CART
 RUNTYP=OPTIMIZE DFTTYP=B3LYP QMTTOL=1.0E-06 MPLEVL=0 $END
 $STATPT NSTEP=400 OPTTOL=0.0001 $END
 $SCF DIRSCF=.T. SHIFT=.T. DAMP=.T. DIIS=.T. CONV=1.0d-04 $END
# $DFT DFTTYP=B3LYP METHOD=GRID $END
 $BASIS GBASIS=N31 NGAUSS=6 NDFUNC=1 NPFUNC=0 DIFFSP=.F. DIFFS=.F. $END
 $DATA
DMS:/B3LYP/6-31++G(d,p)/acn
C1
C    6.0    0.68659   1.19949  -0.00622
C    6.0   -0.70313   1.21419  -0.00025
C    6.0   -1.41775   0.01743   0.00241
C    6.0   -0.72317  -1.19015  -0.00025
C    6.0    0.67066  -1.20182  -0.00567
C    6.0    1.39935  -0.00785  -0.00668
H    1.0    1.22766   2.14042  -0.01545
H    1.0   -1.24623   2.15171   0.00174
H    1.0   -1.26565  -2.13139   0.00246
H    1.0    1.19419  -2.15250  -0.01428
O    8.0   -2.79223   0.09349   0.00961
H    1.0   -3.16377  -0.79407   0.00208
N    7.0    2.80400  -0.01587  -0.07606
H    1.0    3.24865   0.81309   0.29217
H    1.0    3.23975  -0.85185   0.28685
 $END
```

Input file for Tetraphenylporphyrin

```
$SYSTEM TIMLIM=600000.0 MEMORY=51200000 $END
 $CONTRL SCFTYP=RHF MPLEVL=0 MAXIT=200 ICHARG=0 MULT=1 COORD=CART
 RUNTYP=OPTIMIZE DFTTYP=B3LYP QMTTOL=1.0E-06 MPLEVL=0 $END
 $STATPT NSTEP=400 OPTTOL=0.0001 $END
 $SCF DIRSCF=.T. SHIFT=.T. DAMP=.T. DIIS=.T. CONV=1.0d-04 $END
# $DFT DFTTYP=B3LYP METHOD=GRID $END
 $BASIS GBASIS=N31 NGAUSS=6 NDFUNC=1 NPFUNC=0 DIFFSP=.F. DIFFS=.F. $END
 $DATA
C1
C    6.0    1.36337   2.78531   0.01344
C    6.0    1.02475   4.17834   0.00310
C    6.0   -0.33925   4.28546  -0.00075
C    6.0   -0.89108   2.96275   0.00492
N    7.0    0.17488   2.09410   0.01669
C    6.0    2.65093   2.23228   0.00537
C    6.0   -2.24943   2.62140  -0.01130
C    6.0   -2.76342   1.31087  -0.01458
C    6.0   -4.19233   1.01588  -0.04271
C    6.0   -4.30382  -0.33217  -0.03901
C    6.0   -2.94265  -0.85747  -0.00601
N    7.0   -2.03185   0.15903   0.00460
C    6.0   -2.64821  -2.23420   0.00120
C    6.0   -1.36061  -2.78745   0.00192
C    6.0   -1.02196  -4.17990   0.02860
C    6.0    0.34220  -4.28694   0.02489
C    6.0    0.89367  -2.96430  -0.00144
N    7.0   -0.17231  -2.09601  -0.01626
C    6.0    2.25222  -2.62303  -0.00513
C    6.0    2.76640  -1.31270  -0.01155
C    6.0    4.19555  -1.01791  -0.00760
C    6.0    4.30741   0.32998   0.00308
C    6.0    2.94599   0.85564  -0.00707
N    7.0    2.03482  -0.16056  -0.01290
C    6.0    3.79699   3.20237   0.01279
C    6.0    4.49924   3.48464  -1.16470
C    6.0    5.55713   4.39272  -1.16135
C    6.0    5.92735   5.03145   0.02099
C    6.0    5.23574   4.75629   1.19942
C    6.0    4.17678   3.84918   1.19417
H    1.0    1.74354   4.98123  -0.00417
H    1.0   -0.92429   5.19067  -0.01141
H    1.0   -4.98563   1.74632  -0.06758
H    1.0   -5.20633  -0.92244  -0.06239
H    1.0   -1.74048  -4.98272   0.05344
H    1.0    0.92736  -5.19184   0.04521
H    1.0    4.98918  -1.74846  -0.00543
H    1.0    5.21041   0.91980   0.01551
H    1.0    4.21117   2.99084  -2.08616
H    1.0    6.08919   4.60296  -2.08255
H    1.0    5.52052   5.24597   2.12407
```

H	1.0	3.64116	3.63509	2.11243
C	6.0	-3.22553	3.76302	-0.03116
C	6.0	-3.70809	4.31097	1.16253
C	6.0	-3.66945	4.29802	-1.24581
C	6.0	-4.61749	5.36773	1.14278
H	1.0	-3.37022	3.90311	2.10886
C	6.0	-4.57919	5.35430	-1.26692
H	1.0	-3.30037	3.88052	-2.17609
C	6.0	-5.05579	5.89172	-0.07224
H	1.0	-4.98343	5.78009	2.07665
H	1.0	-4.91474	5.75658	-2.21645
H	1.0	-5.76334	6.71318	-0.08801
C	6.0	3.22821	-3.76491	0.00698
C	6.0	3.71848	-4.27045	1.21645
C	6.0	3.66176	-4.34478	-1.19042
C	6.0	4.62483	-5.32976	1.22845
H	1.0	3.38796	-3.82802	2.14972
C	6.0	4.56882	-5.40370	-1.17980
H	1.0	3.28724	-3.96019	-2.13268
C	6.0	5.05256	-5.89900	0.02999
H	1.0	4.99612	-5.70954	2.17395
H	1.0	4.89659	-5.84073	-2.11662
H	1.0	5.75786	-6.72248	0.03888
C	6.0	-3.79438	-3.20367	0.01381
C	6.0	-4.49405	-3.46316	1.19785
C	6.0	-4.18442	-3.86454	-1.15686
C	6.0	-5.55893	-4.36284	1.21193
H	1.0	-4.19923	-2.95647	2.11007
C	6.0	-5.25070	-4.76266	-1.14448
H	1.0	-3.65243	-3.66661	-2.08086
C	6.0	-5.94069	-5.01451	0.04031
H	1.0	-6.08912	-4.55402	2.13842
H	1.0	-5.54343	-5.26267	-2.06120
H	1.0	-6.76994	-5.71300	0.05031
H	1.0	-0.09621	-1.08595	-0.02220
H	1.0	0.09840	1.08407	0.02253
H	1.0	6.75013	5.73725	0.02439

$END

Input file for **5b**

```
$SYSTEM TIMLIM=600000.0 MEMORY=51200000 $END
 $CONTRL SCFTYP=RHF MPLEVL=0 MAXIT=200 ICHARG=0 MULT=1 COORD=CART
 RUNTYP=OPTIMIZE DFTTYP=B3LYP QMTTOL=1.0E-06 MPLEVL=0 $END
 $STATPT NSTEP=400 OPTTOL=0.0001 $END
 $SCF DIRSCF=.T. SHIFT=.T. DAMP=.T. DIIS=.T. CONV=1.0d-04 $END
# $DFT DFTTYP=B3LYP METHOD=GRID $END
 $BASIS GBASIS=N31 NGAUSS=6 NDFUNC=1 NPFUNC=0 DIFFSP=.F. DIFFS=.F. $END
 $DATA
DMS:/B3LYP/6-31++G(d,p)/acn
CN 1

C   6.0  -6.058288  -2.806362   7.872034
C   6.0  -5.370036  -4.173284   7.575187
C   6.0  -4.269261  -4.212230   6.733858
C   6.0  -4.317106  -2.914990   6.248166
N   7.0  -5.063229  -2.055086   7.139670
C   6.0  -7.380109  -2.389462   7.872034
C   6.0  -3.736519  -2.499647   5.060131
C   6.0  -3.836278  -1.174564   4.666150
C   6.0  -3.292818  -0.659268   3.499900
C   6.0  -3.559769   0.695837   3.383949
C   6.0  -4.294562   1.151793   4.467093
N   7.0  -4.461744  -0.251237   5.336894
C   6.0  -4.410467   2.512766   4.702332
C   6.0  -5.373848   2.984789   5.579891
C   6.0  -5.571425   4.322680   5.883253
C   6.0  -6.852684   4.232088   7.193619
C   6.0  -6.909360   2.862494   7.398601
N   7.0  -6.314013   2.147772   6.291512
C   6.0  -7.472078   2.257942   8.511652
C   6.0  -7.488710   0.875935   8.615559
C   6.0  -8.118640   0.172482   9.630133
C   6.0  -8.954939  -0.723930   8.316916
C   6.0  -7.679974  -1.036282   7.872034
N   7.0  -6.923949   0.066159   7.767652
C   6.0  -3.570840   3.402582   4.050973
C   6.0  -3.066376  -3.411858   4.260259
C   6.0  -8.402027  -3.325708   7.884036
C   6.0  -8.028789   3.035080   9.515219
C   6.0  -2.976829  -4.739384   4.648426
C   6.0  -2.316933  -5.653976   3.842774
C   6.0  -1.746583  -5.241042   2.648955
C   6.0  -1.836127  -3.913566   2.260802
C   6.0  -2.496017  -2.998952   3.066434
C   6.0  -2.615329   2.931421   3.164393
C   6.0  -1.783625   3.822062   2.504056
C   6.0  -1.907432   5.183863   2.730298
C   6.0  -2.862907   5.655007   3.616845
C   6.0  -3.694624   4.764389   4.277196
C   6.0  -8.022774   4.416743   9.405725
C   6.0  -8.590075   5.193480  10.403657
```

C	6.0	-9.163390	4.588554	11.511082
C	6.0	-9.169405	3.206943	11.620572
C	6.0	-8.602111	2.430181	10.622656
C	6.0	-9.723858	-2.908838	7.884037
C	6.0	-10.745794	-3.845141	7.884037
C	6.0	-10.445899	-5.198315	7.884037
C	6.0	-9.124118	-5.615171	7.884036
C	6.0	-8.102162	-4.678889	7.884036
C	6.0	-3.113099	1.574722	2.200871
N	7.0	-2.382853	0.767756	1.248788
C	6.0	-1.856365	1.369910	0.044158
C	6.0	-0.493073	1.344695	-0.204473
C	6.0	0.010337	1.911386	-1.364843
C	6.0	-0.849546	2.503292	-2.276581
C	6.0	-2.212786	2.528506	-2.027960
C	6.0	-2.716223	1.961823	-0.867598
O	8.0	-0.338689	3.069080	-3.462760
C	6.0	1.061998	2.910433	-3.494673
C	6.0	1.619957	3.528388	-4.790220
C	6.0	3.149789	3.355113	-4.825076
C	6.0	3.707748	3.973068	-6.120622
C	6.0	5.237580	3.799793	-6.155478
C	6.0	5.795539	4.417748	-7.451024
C	6.0	7.325372	4.244473	-7.485880
C	6.0	7.883330	4.862428	-8.781427
C	6.0	9.413163	4.689153	-8.816283
C	6.0	9.971122	5.307108	-10.111829
C	6.0	10.632514	4.550911	-10.974836
H	1.0	-5.809731	-5.086797	8.055845
H	1.0	-3.456588	-4.889063	6.359249
H	1.0	-4.900624	-1.045680	7.246499
H	1.0	-2.722600	-1.261561	2.744277
H	1.0	-5.154659	5.295991	5.511982
H	1.0	-7.545711	4.859773	7.813777
H	1.0	-6.523345	1.170958	6.049093
H	1.0	-8.349633	-0.078706	10.698972
H	1.0	-9.981826	-0.966440	7.935389
H	1.0	-3.446838	-5.075565	5.610166
H	1.0	-2.252766	-6.730505	4.152351
H	1.0	-1.220727	-5.983308	1.992154
H	1.0	-1.382753	-3.581232	1.289783
H	1.0	-2.576811	-1.926263	2.747560
H	1.0	-2.521538	1.829685	2.973994
H	1.0	-1.016583	3.441286	1.779124
H	1.0	-1.240610	5.905445	2.188506
H	1.0	-2.969547	6.757988	3.792718
H	1.0	-4.474517	5.146431	4.987605
H	1.0	-7.567234	4.906093	8.504676
H	1.0	-8.593755	6.311587	10.310417
H	1.0	-9.631160	5.216983	12.314287
H	1.0	-9.642047	2.716948	12.512416
H	1.0	-8.615541	1.311420	10.706706
H	1.0	-9.966618	-1.813464	7.874320

```
H   1.0  -11.815795   -3.507690    7.874321
H   1.0  -11.273141   -5.956239    7.874320
H   1.0   -8.881370   -6.710547    7.874320
H   1.0   -7.032168   -5.016363    7.874320
H   1.0   -2.452809    2.400584    2.576155
H   1.0   -4.015097    2.018032    1.702125
H   1.0   -2.245602   -0.235832    1.424071
H   1.0    0.201995    0.857048    0.528979
H   1.0    1.112906    1.882466   -1.570692
H   1.0   -2.909832    2.999140   -2.770591
H   1.0   -3.820784    1.973738   -0.670942
H   1.0    1.316062    1.818099   -3.461080
H   1.0    1.517515    3.425076   -2.607816
H   1.0    1.365893    4.620722   -4.823813
H   1.0    1.164439    3.013744   -5.677077
H   1.0    3.403853    2.262779   -4.791482
H   1.0    3.605307    3.869756   -3.938218
H   1.0    3.453684    5.065402   -6.154216
H   1.0    3.252230    3.458424   -7.007480
H   1.0    5.491644    2.707459   -6.121884
H   1.0    5.693098    4.314437   -5.268621
H   1.0    5.541475    5.510082   -7.484618
H   1.0    5.340022    3.903104   -8.337882
H   1.0    7.579435    3.152139   -7.452287
H   1.0    7.780889    4.759117   -6.599023
H   1.0    7.629266    5.954762   -8.815020
H   1.0    7.427813    4.347784   -9.668284
H   1.0    9.667227    3.596819   -8.782689
H   1.0    9.868680    5.203797   -7.929425
H   1.0    9.825649    6.399976  -10.320029
H   1.0   11.039023    5.001130  -11.918723
H   1.0   10.777987    3.458043  -10.766636
$END
```

Input file for **5b**-congener that has alkenyl group at the porphyn ring

```
$SYSTEM TIMLIM=600000.0 MEMORY=51200000 $END
 $CONTRL SCFTYP=RHF MPLEVL=0 MAXIT=200 ICHARG=0 MULT=1 COORD=CART
 RUNTYP=OPTIMIZE DFTTYP=B3LYP QMTTOL=1.0E-06 MPLEVL=0 $END
 $STATPT NSTEP=400 OPTTOL=0.0001 $END
 $SCF DIRSCF=.T. SHIFT=.T. DAMP=.T. DIIS=.T. CONV=1.0d-04 $END
# $DFT DFTTYP=B3LYP METHOD=GRID $END
 $BASIS GBASIS=N31 NGAUSS=6 NDFUNC=1 NPFUNC=0 DIFFSP=.F. DIFFS=.F. $END
 $DATA
DMS:/B3LYP/6-31++G(d,p)/acn

C1
C   6.0    4.18044    0.75782    0.13186
C   6.0    4.37062   -0.52929   -0.10911
C   6.0    2.88782   -0.96559   -0.10602
N   7.0    2.06648    0.07975   -0.01408
C   6.0    2.85024    1.20565    0.10307
C   6.0   -0.60399    4.09150   -0.16199
C   6.0    0.71566    4.11189   -0.28132
C   6.0    1.14754    2.85793    0.02466
N   7.0    0.04313    2.01604    0.08591
C   6.0   -1.05852    2.82602    0.11320
C   6.0    2.51017    2.50571    0.18505
C   6.0   -4.25164   -0.67533    0.27685
C   6.0   -4.35197    0.63540    0.11165
C   6.0   -2.93864    1.21721    0.14846
N   7.0   -2.10903    0.07321    0.07278
C   6.0   -2.86183   -0.99712    0.16886
C   6.0   -2.42589    2.50764    0.27094
C   6.0    0.53445   -4.05015   -0.20454
C   6.0   -0.74720   -4.02924   -0.25763
C   6.0   -1.20993   -2.71851   -0.00896
N   7.0   -0.07359   -1.95158    0.04871
C   6.0    1.02271   -2.71835   -0.04850
C   6.0    2.33235   -2.45886   -0.08430
C   6.0   -2.51687   -2.46589    0.11616
C   6.0   -3.22197    3.84866    0.22025
C   6.0    3.51512    3.68556    0.25171
C   6.0    2.98435   -3.78199   -0.11871
C   6.0   -3.41852   -3.69499    0.09399
C   6.0   -4.30827    3.57269   -0.25424
C   6.0   -4.99853    4.30731   -1.35429
C   6.0   -4.84033    5.61966   -1.36163
C   6.0   -3.90098    6.11274   -0.46442
C   6.0   -3.10998    5.21439    0.29593
C   6.0    3.18384    4.98946    0.67929
C   6.0    3.87806    6.11439    0.19949
C   6.0    5.16224    5.94221   -0.26470
C   6.0    5.64224    4.65491   -0.37142
```

C	6.0	4.76868	3.59506	-0.33914
C	6.0	4.22722	-3.63384	-0.36130
C	6.0	4.75878	-4.37419	-1.35734
C	6.0	4.57030	-5.72002	-1.14847
C	6.0	3.79559	-6.07300	-0.08045
C	6.0	2.91030	-5.11570	0.33967
C	6.0	-3.12994	-4.98852	0.46901
C	6.0	-3.85562	-6.09225	0.05300
C	6.0	-5.15981	-5.89212	-0.31711
C	6.0	-5.61839	-4.60006	-0.44697
C	6.0	-4.70859	-3.56879	-0.47292
C	6.0	-5.85665	0.93472	-0.04060
N	7.0	-6.33497	2.20639	-0.45761
C	6.0	-7.01624	3.19195	0.40938
C	6.0	-8.31452	3.05909	0.05635
C	6.0	-8.98625	4.19185	-0.40867
C	6.0	-8.49930	5.50091	-0.23830
C	6.0	-7.34965	5.71539	0.47039
C	6.0	-6.62769	4.56174	0.84909
O	8.0	-9.09247	6.55852	-0.85029
C	6.0	5.89604	-0.84417	-0.10463
C	6.0	6.24387	-2.27457	0.10949
C	6.0	7.37132	-3.31002	0.12824
C	6.0	6.98138	-4.86874	0.19783
C	6.0	7.89446	-6.06435	0.07695
C	6.0	7.13327	-7.43424	-0.03316
C	6.0	8.23125	-8.44351	-0.15946
C	6.0	7.72778	-9.86091	-0.17926
C	6.0	8.96734	-10.72643	-0.23939
C	6.0	8.58211	-12.18522	-0.22282
C	6.0	9.83239	-13.03318	-0.27004
H	1.0	5.04199	1.34914	0.34105
H	1.0	-1.09479	5.02107	-0.29649
H	1.0	1.20727	5.04542	-0.45533
H	1.0	0.04980	1.00437	0.11312
H	1.0	-5.10238	-1.30554	0.31308
H	1.0	0.91646	-5.02265	-0.35478
H	1.0	-1.17197	-5.01735	-0.50389
H	1.0	-0.06508	-0.92766	-0.03001
H	1.0	-4.78245	2.95260	0.51308
H	1.0	-5.96901	3.98496	-1.81323
H	1.0	-5.64519	6.22669	-1.81266
H	1.0	-3.82649	7.17658	-0.36308
H	1.0	-2.29918	5.77345	0.82325
H	1.0	2.27891	5.17493	1.25864
H	1.0	3.41684	7.09952	0.18869
H	1.0	5.78544	6.78281	-0.54860
H	1.0	6.68318	4.43880	-0.58564
H	1.0	5.10879	2.70500	-0.84035
H	1.0	4.42020	-2.85616	0.33276
H	1.0	5.65161	-4.03349	-1.87992
H	1.0	5.23768	-6.43526	-1.59162
H	1.0	3.83501	-7.03918	0.40324

H	1.0	2.12519	-5.51269	0.93854
H	1.0	-2.23148	-5.22184	1.04462
H	1.0	-3.43538	-7.09849	0.11083
H	1.0	-5.83072	-6.72048	-0.47556
H	1.0	-6.68203	-4.36746	-0.51930
H	1.0	-5.07718	-2.66693	-0.98091
H	1.0	-6.42746	0.63991	0.93557
H	1.0	-6.21934	0.28752	-0.83659
H	1.0	-5.58059	2.39067	-1.15110
H	1.0	-8.59653	2.06353	-0.28090
H	1.0	-9.80318	3.99940	-1.07230
H	1.0	-6.93464	6.73118	0.52587
H	1.0	-5.65691	4.86361	1.27340
H	1.0	-9.94559	6.26199	-1.20423
H	1.0	6.37290	-0.30903	0.71735
H	1.0	6.32285	-0.50506	-1.06314
H	1.0	5.80024	-2.44874	-0.86895
H	1.0	5.79934	-2.41705	1.10489
H	1.0	7.91743	-3.08137	1.02894
H	1.0	7.88534	-3.12178	-0.81048
H	1.0	6.65537	-5.07333	-0.79766
H	1.0	6.22689	-5.10720	0.96345
H	1.0	8.56745	-6.07662	0.92598
H	1.0	8.45854	-5.96343	-0.86241
H	1.0	6.52486	-7.59784	-0.93174
H	1.0	6.47069	-7.64175	0.81867
H	1.0	8.91521	-8.29459	0.67947
H	1.0	8.77392	-8.23893	-1.09248
H	1.0	7.08102	-10.04448	-1.04662
H	1.0	7.14197	-10.07266	0.72233
H	1.0	9.61636	-10.49243	0.61540
H	1.0	9.53965	-10.48829	-1.14642
H	1.0	7.93992	-12.40684	-1.08215
H	1.0	8.01023	-12.40408	0.68569
H	1.0	10.40370	-12.82394	-1.18038
H	1.0	9.56572	-14.09101	-0.25686
H	1.0	10.47350	-12.81993	0.59171

$END
